# The miR-15a/16-1 and miR-15b/16-2 clusters regulate early B cell development by limiting IL-7 receptor expression

**DOI:** 10.3389/fimmu.2022.967914

**Published:** 2022-08-25

**Authors:** Katharina Hutter, Thomas Rülicke, Tamas G. Szabo, Lill Andersen, Andreas Villunger, Sebastian Herzog

**Affiliations:** ^1^ Institute of Developmental Immunology, Biocenter, Medical University Innsbruck, Innsbruck, Austria; ^2^ Department of Biomedical Sciences, University of Veterinary Medicine Vienna, Vienna, Austria; ^3^ Ludwig Boltzmann Institute for Hematology and Oncology, Vienna, Austria; ^4^ Research Center for Molecular Medicine (CeMM) of the Austrian Academy of Sciences, Vienna, Austria; ^5^ Ludwig Boltzmann Institute for Rare and Undiagnosed Diseases, Vienna, Austria

**Keywords:** B cell development, microRNA, miR-15 family, knockout (KO) mice, IL-7 receptor

## Abstract

MicroRNAs are small non-coding RNAs that have emerged as post-transcriptional regulators involved in development and function of different types of immune cells, and aberrant miRNA expression has often been linked to cancer. One prominent miRNA family in the latter setting is the miR-15 family, consisting of the three clusters miR-15a/16-1, miR-15b/16-2 and miR-497/195, which is best known for its prominent tumor suppressive role in chronic lymphocytic leukemia (CLL). However, little is known about the physiological role of the miR-15 family. In this study, we provide a comprehensive *in vivo* analysis of the physiological functions of miR-15a/16-1 and miR-15b/16-2, both of which are highly expressed in immune cells, in early B cell development. In particular, we report a previously unrecognized physiological function of the miR-15 family in restraining progenitor B cell expansion, as loss of both clusters induces an increase of the pro-B as well as pre-B cell compartments. Mechanistically, we find that the miR-15 family mediates its function through repression of at least two different types of target genes: First, we confirm that the miR-15 family suppresses several prominent cell cycle regulators such as *Ccne1*, *Ccnd3* and *Cdc25a* also *in vivo*, thereby limiting the proliferation of progenitor B cells. Second, this is complemented by direct repression of the *Il7r* gene, which encodes the alpha chain of the IL-7 receptor (IL7R), one of the most critical growth factor receptors for early B cell development. In consequence, deletion of the miR-15a/16-1 and miR-15b/16-2 clusters stabilizes *Il7r* transcripts, resulting in enhanced IL7R surface expression. Consistently, our data show an increased activation of PI3K/AKT, a key signaling pathway downstream of the IL7R, which likely drives the progenitor B cell expansion we describe here. Thus, by deregulating a target gene network of cell cycle and signaling mediators, loss of the miR-15 family establishes a pro-proliferative milieu that manifests in an enlarged progenitor B cell pool.

## Introduction

B cells develop in the bone marrow, where they undergo two sequential steps of immunoglobulin gene segment rearrangements, finally giving rise to a unique B cell receptor (BCR). Heavy chain diversity (D) to joining (J) gene segment rearrangement is initiated between the pre-pro and pro-B cell stage and is followed by variable (V) to DJ gene segment recombination in late pro-B cells (1). Successful rearrangement results in expression of an immunoglobulin heavy chain that pairs with the surrogate light chain components VpreB and lambda5, thereby forming the pre-B cell receptor (pre-BCR) (2). Expression of a functional pre-BCR in pre-B cells induces several rounds of cell division before the cell cycle is stalled and light chain (VJ) recombination is initiated. The thereby formed light chain pairs with the heavy chain to form the BCR, marking the immature B cell stage (3). Immature B cells travel to the spleen where they undergo additional maturation steps through several transitional stages, eventually entering the mature B cell pool as marginal zone or follicular B cells (4, 5).

As heavy and light chain gene segment recombination in early B cell development is based on the introduction of DNA double strand breaks, the stepwise progression through the abovementioned stages needs to be tightly regulated. This ensures the proper separation of proliferation and cell cycle arrest to allow somatic rearrangements, which has been demonstrated as critical to maintain genomic stability and to prevent malignant transformation and blood cancer (6–8). In early B cells, this biphasic sequence is mainly coordinated by the interplay of three receptors and their downstream signaling pathways: The IL-7 receptor (IL7R), which contributes to pro- and pre-B cell survival, is critical for proliferation and at the same time inhibits differentiation by downregulation of FOXO1 and repression of *Rag1* and *Rag2* genes (9); the abovementioned pre-BCR, as well mediating cell survival and proliferation, but also light chain recombination (10); and the chemokine receptor CXCR4, which instructs proper positioning of B cell progenitors in the bone marrow microenvironment and promotes cell cycle exit, pre-BCR repression and light chain rearrangement (11). However, even with these main players identified, it appears fair to say that the precise gene regulatory networks that govern early B cell development are not fully understood.

Since their discovery, microRNAs (miRNAs), small non-coding RNAs with a length of about 21 to 25 nucleotides, have emerged to establish an additional layer of gene regulation in almost all biological processes. They exert their function by sequence-specific binding to the 3’ UTR of target mRNAs, resulting in inhibition of translation or mRNA decay and ultimately in post-transcriptional repression of their target genes (12). Given that more than 60% of all protein coding genes in mammals are predicted to be targeted by miRNAs (13), it is not surprising that also B cell development in the bone marrow is shaped by certain miRNAs such as the miR-17-92 cluster and miR-34a (14, 15). However, with an estimated number of about 50 different miRNAs expressed at functionally relevant levels in early B cell development (16), it is likely that additional regulators will emerge upon thorough analysis, such as in *in vivo* loss-of-function mouse models. A possible candidate in this context is the miR-15 family, comprising the three bicistronic clusters miR-15a/16-1, miR-15b/16-2 and miR-497/195 (17). Of these clusters, only the first two are highly expressed throughout B cell development, whereas miR-497 and miR-195 are predominantly found in non-immune cells and consequently both have been described as dispensable for the immune system in a previous study (18). We therefore decided to focus on miR-15a/16-1 and miR-15b/16-2 for this particular study. The miR-15 family is best known for its tumor-suppressive activity in B cell chronic lymphocytic leukemia (CLL), in which the chromosomal region encoding the miR-15a/16-1 cluster is deleted in about 60% of all patients (19). This role has been recapitulated in mouse models, in which deletion of either the miR-15a/16-1 or the miR-15b/16-2 cluster also promoted a CLL-like disease (20, 21). However, both mouse models have been reported to display normal B cell development, possibly indicating that the lack of obvious phenotypes in individual miR-15a/16-1 and miR-15b/16-2 knockout mice is the result of a compensatory effect by the remaining cluster. In fact, our own work using an *in vitro* knockdown approach that targets the whole miR-15 family suggested that the miR-15 family fine-tunes the pre-B to immature B cell transition (22).

To investigate this in detail and in a complex *in vivo* environment, we have therefore generated a conditional mouse model for the immune cell-specific deletion of both miR-15a/16-1 and miR-15b/16-2 clusters. Exploiting this model, we demonstrate that one of the main physiological functions of the miR-15 family is to restrict expansion of B cell progenitors in a dose-dependent manner. Mechanistically, our data show that the miR-15 family directly limits expression of the IL7R, and that loss of this repression results in increased IL7R protein levels as well as in enhanced activation of the PI3K/AKT signaling pathway. Thus, in synergy with the repression of cell-cycle regulatory genes that have been previously described to establish an anti-proliferative state in various systems, such as *Cdc25a*, *Ccnd3* and *Ccne1*, the miR-15 family constricts growth factor receptor signaling and thereby contributes to the biphasic sequence with concomitant phases of proliferation and cell cycle arrest.

## Material and methods

### Animals

The conditional miR-15a/16-1 and miR-15b/16-2 alleles were generated by CRISPR/Cas9-facilitated homologous recombination in murine ES cells, as already described for targeting of the miR-497/195 cluster (18). In short, KH2 ES cells [Premsrirut et al. (23), C57BL/6 x 129/Sv background, kindly provided by J. Zuber, IMP, Vienna] were electroporated (Nucleofector Kit; Lonza, Switzerland) with two Cas9/sgRNA vectors encoding GFP [pX458, Addgene #48138, kindly provided by Feng Zhang (24)] as a marker and the targeting DNA template containing the miRNA cluster flanked by loxP sites (see **Supplementary Table S1** for detailed sgRNA sequences). After 36 h, ES cells were sorted for GFP^hi^ cells and plated at a low density on feeder cells. Individual ES cell clones were screened by PCR, sequenced, and then used for injection in C57BL/6NRj blastocysts. High percentage chimeras were bred with C57BL/6NRj females to confirm germline transmission and then further backcrossed to generate a congenic strain. Cre-mediated recombination in hematopoietic stem cells was induced by mating with C57BL/6N.Cg-Tg(Vav-Cre) mice (25) or C57BL/6N.Cg-Tg(Mb1-Cre) mice to mediate B-cell specific deletion (26).

Animals were kept pathogen-free according to FELASA recommendations (27) under controlled environmental conditions (temperature 22°C ± 1°C, relative humidity of 40–60%), a 12:12 h light/dark cycle, in a facility for laboratory rodents. Food (regular mouse diet; Ssniff, Germany) and water were provided *ad libitum*. Mice were maintained in small groups in individually ventilated cages lined with wood shavings as bedding and enriched with nesting material. If not stated otherwise, mice were analyzed at an age of 10–12 weeks. For all experiments, male and female mice were used in comparable numbers.

### Preparation of single-cell suspension

Single-cell suspensions for flow cytometry were obtained by pulpifying spleens through a 70-µm filter. For bone marrow cell suspensions, femurs and tibiae were isolated, ground, and filtered through a 70-µm filter. Lysis of erythrocytes for spleen and blood samples was performed by incubating the cells for 3–5 min in 1 mL lysis buffer (155 mM NH_4_Cl, 10 mM KHCO_3_, 0.1 mM EDTA; pH 7.5). Cells were resuspended and washed in FACS buffer (PBS with 1% FCS). Cells were counted using a Neubauer counting chamber.

### Flow cytometry

Single-cell suspensions were stained in 96-well plates with 30 µL of the antibody cocktails for 20 min at 4°C. Nonspecific antibody binding was blocked by pre-incubating the cells with anti-CD16/31 antibodies in 30 µL FACS buffer for 10 min at 4°C. All centrifugation steps were performed with 530 g for 2 min. For the antibody cocktails, the following antibodies were used: anti-B220-BV510 (BioLegend, 103247, 1:300), anti-CD19-BV605 (BioLegend; 115540, 1:300), anti-AA4.1-PE/Cy7 (BioLegend; 136507, 1:300), anti-CD25-PE (BioLegend; 102007, 1:300), anti-c-Kit-APC (BioLegend; 135108, 1:300), anti-IgM-FITC (BioLegend; 406506, 1:300), anti-IgD-PerCpCy5.5 (BioLegend; 405710, 1:300), anti-NK1.1-APC (BioLegend; 108709, 1:300), anti-gdTCR-PE (BioLegend; 118108, 1:400), anti-CD27-FITC (BioLegend; 124207, 1:300), anti-CD43-PE (BD Biosciences; 7297616, 1:300), anti-NK1.1-PeCy7 (BioLegend; 108714, 1:300), anti-C11b-APC-Cy7 (BioLegend; 101226, 1:300), anti-TCRbeta-PerCPCy5.5 (BioLegend; 109228, 1:300), and anti-B220-FITC (BioLegend; 103206, 1:300), anti-CD11c-Pe/Cy7 (BioLegend; 117317, 1:300), anti-NK1.1-APC (BioLegend; 108710, 1:300), anti-Ly6C-PerCPCy5 (BioLegend; 128012), anti-Ly6G-BV421 (BioLegend; 127627, 1:300), anti-F4/80-biotin (BioLegend; 123105; 1:300), anti-SiglecF-PE (BioLegend; 155505, 1:300), BV605-Streptavidin (BioLegend; 405229; 1:300), CD127-Pe/Cy7 (BioLegend; 135013, 1:300), anti-CD34-FITC (Miltenyi; 130-105-831, 1:300), anti-Sca1-PE (BioLegend; 108107, 1:300), anti-CD16/32-PerCPCy5.5 (BioLegend; 101323, 1:300), anti-c-Kit-BV421 (BioLegend; 105828, 1:300), APC-Cy7-Streptavidin (BioLegend; 405208, 1:400). The lineage cocktail to exclude mature immune cell populations for the hematopoietic progenitors analysis combined anti-B220-bio, anti-Ter119-bio, anti-CD11b-bio, anti-Gr-1-bio, anti-CD3-bio (eBioscience; 13-0031-75, 1:100).

Apoptotic cells were stained using Topro3 (1:10000) for exclusion of dead cells and AnnexinV-FITC (1:10000) prior to flow cytometric analysis.

Flow cytometric analysis was performed on a FACS LSR Fortessa instrument (BD Biosciences), and sorting was performed on a FACS Aria III (BD Biosciences) cell sorter. Data were analyzed using FlowJo software (BD Life Science, USA).

### Intracellular Staining (pAKT)

After the surface staining, cells were washed in 1% BSA/PBS and fixed in 4% PFA/PBS for 15 min at room temperature (RT). After one washing step (1% BSA/PBS) cells were permeabilized in PermBuffer (saponin-based permeabilization and wash buffer; ThermoFisher) for 15min and subsequently stained with pAKT Ser473 (Cell Signaling; 4060, 1:200) or an IgG Isotype control (Cell Signaling; 3900) for 60 min at RT. Cells were washed and incubated with the secondary antibody anti-rabbit IgG-Alexa647 (Cell Signaling; 4414, 1:500) for 30 min at RT.

### MACS of bone marrow B cells for qPCR

Bone marrow cell suspensions were stained with 100 µl anti-B220-biotin antibody solution (BioLegend; 103204, 1:100) and incubated for 20 min at 4°C. Cells were washed in FACS buffer, the pellet was resuspended in 200 µl MACS beads (Invitrogen; MSNB-6002-74; dilution 1:10 in FACS buffer) and incubated for 5 min at RT. The cell/bead suspension was mixed with 3 ml FACS buffer and placed on a magnet for 10 min at RT. FACS buffer was discarded and the cell/bead suspension was washed once more by repeating the previous step. In the last step, the FACS buffer was discarded and the cell/bead suspension was resuspended in 500 µl Trizol (Invitrogen; 15596-018).

### Quantitative real-time PCR

RNA of sorted cells was isolated using the Quick-RNA Microprep Kit according to manufacturer’s instructions (Zymo; R1050). For analysis of miRNA genes, RNA was isolated using Trizol according to manufacturer’s instructions and reverse-transcribed with the miRCURY LNA RT Kit (QIAGEN, Hilden, Germany; 339340) followed by SYBR Green qPCR (miRCURY LNA SYBR Green PCR Kit; QIAGEN; 339356; miRCURY LNA miRNA PCR Assays (339306); see **Supplementary Table S1** for details) using expression of SNORD68 (small nucleolar RNA, C/D box 68) as a reference. For RNA sequencing target validation, RNA was reverse-transcribed using the iScript cDNA synthesis Kit (Bio-rad 1708891) and cDNA samples were analyzed with the Luna Universal qPCR Master Mix (NEB, M3003; see **Supplementary Table S1** for primer sequences). Final quantification was performed using the ΔΔCT method.

### RNA sequencing

RNA sequencing was performed as a service by Novogene (Cambridge, UK). In short, total RNA was isolated using the RNeasy Micro Kit according to manufacturer’s instructions (QIAGEN; 74004) and amplified and reverse transcribed into cDNA using the SMART-seq protocol (Takara), followed by a quality control and library preparation. Paired end sequencing (reads of 150 nt) was performed on a NovaSeq 6000 (Illumina). For data analysis, single end reads were trimmed using Trimmomatic (28), aligned using STAR aligner (29) and counted with FeatureCounts (30). Differential gene expression was analyzed using EdgeR (31, 32).

### Transfections and retroviral transductions

Retroviral supernatants were produced in HEK293T cells. Per sample, 400 ng of DNA for the plasmid of interest, 150 ng of HIT60 and 150 ng pVSVg were diluted in 20 μl IMDM medium (Sigma-Aldrich) and mixed with 20 μl IMDM medium containing 1.2 μl of 1 mg/mL polyethylenimine (PEI, Polysciences). Following a 20 min incubation step at room temperature, the DNA:PEI mix was pipetted into a 24-well tissue culture plate and 230 μl of HEK293T at a density of 6 × 10^5^ cells per ml were plated on top. After 30 – 40 hours viral supernatants were harvested, mixed with polybrene (16 μg/mL final concentration) and used for spin-infection of cells in 1.5 ml reaction tubes or 96-well plates (400 g at 37°C for 90 min).

### 3’ UTR reporter assays

UTRs of interest were amplified by PCR (see **Supplementary Table S1** for details), purified by agarose gel isolation (Monarch DNA Gel Extraction Kit, NEB (T1020L)) and ligated into LMP-destGFP (22) 3′ of the destabilized GFP cDNA *via XhoI* and *ClaI*. The pre-B cell line WK3 was transduced with the respective retroviral supernatants and cells were selected for puromycin resistance by adding puromycin at a concentration of 1 μg/ml for 3–4 days. For the suppression assays, cells were further transduced with a previously described plasmid encoding dsRed as a marker together with the miR-15a/16-1 cluster (22) or an empty vector as a control. For the sponge-mediated derepression of endogenous miRNAs, a scrambled sponge or a sponge containing multiple binding sites for miR-16 were used (22). In experimental settings, cells were always cultured in the absence of puromycin.

### Cell lines and cell culture

The pre-B cell line WK3 was derived by extended culture of total bone marrow of SLP-65-deficient mice in IL-7-supplemented medium (22). Pre-B cells were cultured in IMDM (Sigma) containing 7.5% FCS (Biochrom Superior), 100 U/ml penicillin, 100 U/ml streptomycin (PAN), 50 μM 2-mercaptoethanol and IL-7 (produced and secreted by transgenic J558L cells) in excess. In short, each batch of the IL-7-containg J558L supernatant was titrated to determine the lowest concentration to support maximal pre-B cell proliferation, and this amount was doubled for standard cell culture. HEK293T cells were cultured in the same medium, but without addition of IL-7.

For the culture of primary B cell progenitors, the bone marrow of miR-15a/b^+/+ Vav-Cre^ or miR-15a/b^fl/fl Vav-Cre^ mice was sorted for the c-KIT^+^B220^+^CD19^-^NK1.1^-^ population and cells were seeded in a 96-well plate in 100 µl IMDM medium (7.5% FCS (Biochrom Superior), 100 U/ml penicillin, 100 U/ml streptomycin (PAN), 50 μM 2-mercaptoethanol, 100 ng/ml SCF, 5 ng/ml IL-7 and 50 ng/ml FLT3L) at a density of 5 x 10^4^ cells/ml. Upon a “recovery phase” of 4-5 days, progenitor cells typically started to proliferate and were subsequently kept in culture and split regularly. B cell progenitors were used for experiments while in this proliferative phase, and were discarded when the proliferation rate dropped significantly, typically after 3-4 weeks of culture. To induce differentiation of progenitors *in vitro*, cells were cultured in the absence of IL-7 for 48 hours.

### EdU assay

To assess *in vitro* proliferation, cells were analyzed with the Click-iT Flow Cytometry Assay Kit (Invitrogen; C10419) according to the manufacturer’s protocol, using a labeling time of 4 hours at an EdU concentration of 10 µg/ml.

### Western blot

For Western blot analysis, cells were lysed in ice-cold RIPA buffer (50 mM Tris–HCl, pH 7.4, 1% NP-40, 0.25% sodium deoxycholate, 150 mM NaCl, 1 mM EDTA (pH 8), protease inhibitor cocktail Sigma, 10 mM Na3VO4, 10 mM NaF), mixed 1:1 with reducing sample buffer (62.5 mM Tris pH 6.8, 2% SDS, 10% Glycerol, 100 mM DTT), boiled for 5 min at 95°C, and loaded onto a SDS–PAGE gel. Western blotting was performed using PVDF membranes.

Proteins were detected using anti-pAKT Ser473 (Cell Signaling; 4060), anti-AKT (Cell Signaling; C67E7), and anti-actin antibodies (Cell Signaling; 13E5). Bound antibodies were visualized with HRP-labeled secondary antibodies and the ECL system (Advansta) on a light-sensitive film (Amersham, GE).

### Statistical analysis

Values in figures depict mean ± standard deviation (SD), and each mouse or experiment is represented as a dot. The two experimental groups were statistically analyzed by unpaired two-tailed Student’s t-tests using Prism 9 software (GraphPad). P-values < 0.05 were considered as statistically significant, and graphs were labeled according to following scheme: ***P < 0.005, **P < 0.005, and *P < 0.05.

## Results

### Generation of conditional miR-15a/16-1 and miR-15b/16-2 double knockout mice

**Figure 1:**
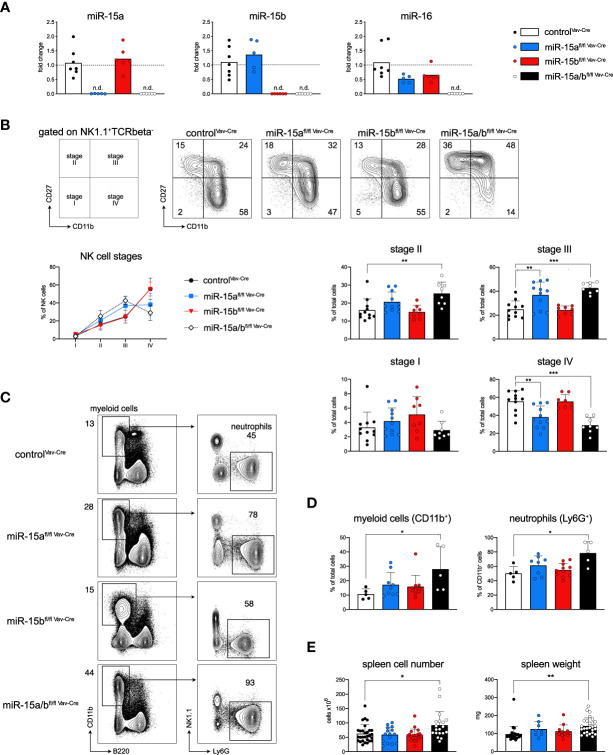
Hematopoietic deletion of miR-15a/16-1 and miR-15b/16-2 blocks NK cell development and promotes the accumulation of myeloid cells. **(A)** Quantitative PCR for the expression of mature miR-15a, miR-15b and miR-16 in B cells derived from miR-15a/b^+/+ Vav-Cre^, miR-15a^fl/fl Vav-Cre^, miR-15b^fl/fl Vav-Cre^ and miR-15a/b^fl/fl Vav-Cre^ mice. Each dot represents the data derived from one mouse. n.d. = not detected. **(B)** NK cells defined as NK1.1^+^TCRbeta^-^ were classified as stage I to stage IV based on their expression of CD27 versus CD11b (schematic left panel and contour plots, numbers indicate the percentage of cells within the respective quadrant). The line and bar graphs show the analysis of all data sets for the depicted genotypes, and again each dot represents data from one mouse. **(C)** Schematic illustration of the gating strategy and representative primary data for myeloid cells and neutrophils in spleens derived from all genotypes. Numbers indicate the percentage of cells within the respective gate. **(D)** Bar graphs show the percentages of indicated populations within total cells or CD11b^+^ myeloid cells for all analyzed mice. **(E)** Bar graphs indicate the total splenic cell numbers and spleen weight for the respective genotypes. Each dot represents the data derived from one mouse. Error bars depict the standard deviation of the mean. Sample groups were statistically compared as indicated by an unpaired two-tailed Student’s t-test. *P < 0.05; **P < 0.005; ***P < 0.0005.

To study whether the miR-15 family is involved in early B cell development, we generated a conditional mouse model for the Cre-mediated deletion of both the miR-15a/16-1 and miR-15b/16-2 clusters, the main family members expressed within the hematopoietic system. The conditional alleles were generated using CRISPR/Cas9-assisted editing to flank the endogenous clusters - embedded within introns of non-coding (*Dleu2* for miR-15a/16-1) and coding (*Smc4* for miR-15b/16-2) genes, respectively - with loxP sites in embryonic stem (ES) cells (**Figures S1A, B**). To delete the respective miRNA clusters in the hematopoietic system, mice derived from these ES cells were intercrossed and then initially bred with the *Vav*-Cre strain (25) to generate single miR-15a/16-1 fl/fl (hereafter miR-15a^fl/fl Vav-Cre^) and miR-15b/16-2 fl/fl (miR-15b^fl/fl Vav-Cre^) as well as miR-15a/16-1 fl/fl miR-15b/16-2 fl/fl (miR-15a/b^fl/fl Vav-Cre^) double knockout (DKO) mice. To verify the loss of the respective miRNAs in all genotypes compared to the control, we analyzed their expression in sorted bone marrow B cells by quantitative PCR. As expected, expression of miR-15a was undetectable in miR-15a^fl/fl Vav-Cre^ and miR-15a/b^fl/fl Vav-Cre^ DKO mice, and likewise there was no miR-15b in miR-15b^fl/fl Vav-Cre^ and DKO mice, respectively. Moreover, since the mature forms miR-16-1 and miR-16-2 are identical, a complete loss of miR-16 was only detectable in miR-15a/b^fl/fl Vav-Cre^ DKO mice, whereas miR-15a^fl/fl Vav-Cre^ and miR-15b^fl/fl Vav-Cre^ mice showed an approximately 50% reduction compared to miR-15a/b^+/+ Vav-Cre^ controls (**Figure 1A**).

### Loss of miR-15 interferes with NK cell development and slightly enhances myeloid cell numbers

To validate the deletion of the respective miR-15 family members also on the functional level, we furthermore compared our mouse model to previously published miR-15 loss-of-function phenotypes. A study by Sullivan et al. has described a partial block in NK cell development at the stage III to stage IV transition upon NK-cell specific deletion of the miR-15a/16-1 cluster (33). Our analysis clearly recapitulated this finding in miR-15a^fl/fl Vav-Cre^ mice, and interestingly the developmental block was further aggravated in miR-15a/b^fl/fl Vav-Cre^ DKO compared to the single knockout mice (**Figures 1B**, **S1C**). However, while clearly contributing to the DKO phenotype, the miR-15b/16-2 cluster alone seemed to be dispensable for NK cell maturation, as miR-15b^fl/fl Vav-Cre^ mice did not show any perturbations upon deletion of the cluster, pointing towards different roles of the individual miR-15 family clusters in this setting (**Figure 1B**). In the myeloid compartment, a recent publication has revealed that concomitant loss of both miR-15a/16-1 as well as miR-15b/16-2, albeit not in a conditional model, promotes the development of acute myeloid leukemia (AML) at an age of approximately 5 months (34). According to our analysis, a dose-dependent myeloid expansion was already detectable in 10 to 12 weeks old mice, with the strongest impact in the DKOs and mainly affecting neutrophils (**Figure 1C, D**). Furthermore and in consequence of the myeloid phenotype, both with respect to relative frequencies and total cell numbers, spleen weight and splenic cellularity were significantly increased in miR-15a/b^fl/fl Vav-Cre^ mice (**Figures 1E**, **S1B**). Together, these data demonstrate a dose-dependent role of the miR-15a/16-1 and miR-15b/16-2 clusters in previously reported non-B cell hematopoietic lineages, and overall validate the function of our mouse model. This, of course, raised the question about the effects of their combined deletion in B cells as the lineage mostly affected by pathological loss of one of the miR-15 family clusters.

### Loss of the miR-15a/16-1 and miR-15b/16-2 clusters impairs B cell development

**Figure 2:**
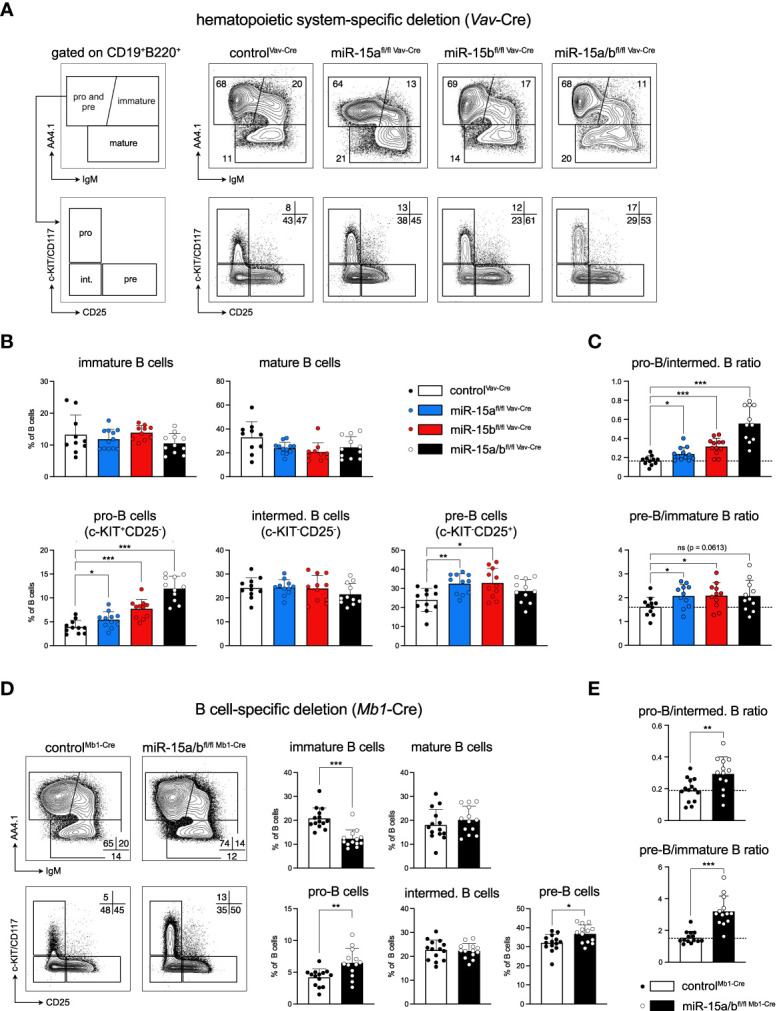
Loss of the miR-15a/16-1 and miR-15b/16-2 cluster impairs early B cell development. **(A)** Representative illustration of the gating strategy for pro-B and pre-B (AA4.1^+^IgM^-^), immature (AA4.1^+^IgM^+^) and mature B cells (AA4.1^-^) in the bone marrow. Pro-B and pre-B cells were further separated into pro-B (c-KIT^+^), intermediate B (c-KIT^-^CD25^-^) and pre-B cells (CD25^+^). The panel on the right provides representative FACS plots for all genotypes analyzed here. Numbers indicate the percentage of cells within the respective gate. **(B)** Bar graphs show the percentage of immature and mature B cells (upper lane) and pro-B, intermediate and pre-B cells (lower lane) within the B220^+^CD19^+^ B cell population in the bone marrow of miR-15a/b^+/+ Vav-Cre^, miR-15a^fl/fl Vav-Cre^, miR-15b^fl/fl Vav-Cre^ and miR-15a/b^fl/fl Vav-Cre^ mice. Each dot represents the data derived from one mouse. **(C)** Ratio of the relative percentages of pro-B to intermediate B cells as well as pre-B to immature B cells calculated from the data in **(B)**. A dashed line marks the averaged ratio of the control^Vav-Cre^ mice. Each dot represents the data derived from one animal. **(D)** Representative FACS plots and bar graphs show the percentage of B cell subpopulations in the bone marrow upon B cell-specific deletion (*Mb1-Cre*) of the miR-15a/16-1 and miR-15b/16-2 clusters. **(E)**. As in C, bar graphs depict the pro-B to intermediate B cell ratio as well as the pre-B to immature B cell ratio for control^Mb1-Cre^ mice and miR-15a/b^fl/fl Mb1-Cre^ mice. A dashed line marks the averaged ratio of the control. Error bars depict the standard deviation of the mean. Each of the different miR-15 knockout groups were statistically compared to control mice by an unpaired two-tailed Student’s t-test. *P < 0.05; **P < 0.005; ***P < 0.0005.

Motivated by our previous *in vitro* study pointing towards a role of the miR-15 family in early B cell development (Lindner et al., 2017), we analyzed the B cell composition of the bone marrow in miR-15 single and DKO mice (**Figure 2A**). Overall, the total number of B cells in the bone marrow was reduced upon miR-15 loss, which is likely caused by the expansion of the myeloid cell pool (**Figure S2A**). Within the B cell compartment, we observed a dose-dependent increase in the pro-B cell subset for all three knockout genotypes (**Figure 2B**), with the effect being most severe in the DKO mice. Notably and as discussed later, we also observed a significantly higher surface expression of the pro-B cell marker c-KIT/CD117, in response to transcriptional upregulation of the respective gene, in mice lacking miR-15 expression (**Figures 2A**, **S2B**). In analogy to the pro-B cells, the pre-B cell fractions were increased in the single knockouts, but not significantly in the DKO, most likely due to the strong relative increase in pro-B cells (**Figure 2B**). In contrast, the intermediate B cell population (c-KIT^-^CD25^-^), an in-between stage cells pass through during differentiation from pro- (c-KIT^+^CD25^-^) to pre-B cells (c-KIT^-^CD25^+^), was not significantly altered in any of the genotypes. Notably, determining the ratio of pro-B to intermediate B cells revealed a dose-dependent shift towards pro-B cells upon loss of the miR-15 family, pointing towards a compromised transition at this stage (**Figure 2C**, upper panel). Likewise, pre-B cells in all knockout genotypes were relatively enriched over immature B cells (**Figure 2C**, lower panel), indicating a mild developmental block. To test whether the observed effect of the miR-15 family on early B cell development is B cell intrinsic, we additionally generated a B cell-specific deletion of both miR-15a/16-1 and miR-15b/16-2 clusters. Here, we made use of the *Mb1*-Cre strain in which Cre is expressed already at a very early pro-B cell stage, enabling us to study the effect of the combined loss of the miR-15a/b clusters at the earliest possible time point (26). In accordance with the miR-15a/b^fl/fl Vav-Cre^ mice, the B cell-specific deletion of miR-15a/b resulted in elevated pro-B cell frequencies in miR-15a/b^fl/fl Mb1-Cre^ mice (**Figures 2D**, **S2C**), indicating that the early B cell phenotype is indeed B cell intrinsic. Notably, pre-B cells were also significantly increased in this system, and the ratio of pro- to intermediate B cells as well of pre- to immature B cells again revealed developmental blocks, albeit incomplete, at the pro- and the pre-B cell stages (**Figures 2C, D**, **S2C**). This suggests that both pro-B as well as pre-B fractions expand upon loss of the miR-15 family, and furthermore confirms our previous *in vitro* data showing an involvement of the miR-15 family in the pre-B to immature B cell transition (22).

To exclude that the higher progenitor B cell percentages are derived from a general bias of hematopoiesis towards the lymphocyte lineage, we examined the bone marrow of *Vav*-Cre mice regarding its composition of different hematopoietic lineage progenitors, i.e. common lymphoid progenitors (CLPs), granulocyte-monocyte progenitors (GMPs), megakaryocyte-erythroid progenitors (MEPs) and common myeloid progenitors (CMPs). However, these subsets did not show any alterations in miR-15a^fl/fl Vav-Cre^, miR-15b^fl/fl Vav-Cre^ and miR-15a/b^fl/fl Vav-Cre^ mice (**Figure S3**), indicating that the miR-15 family affects cells only once committed to the B lymphocyte lineage.

### Loss of the miR-15 family results in pro-B cell expansion *in vitro*

**Figure 3:**
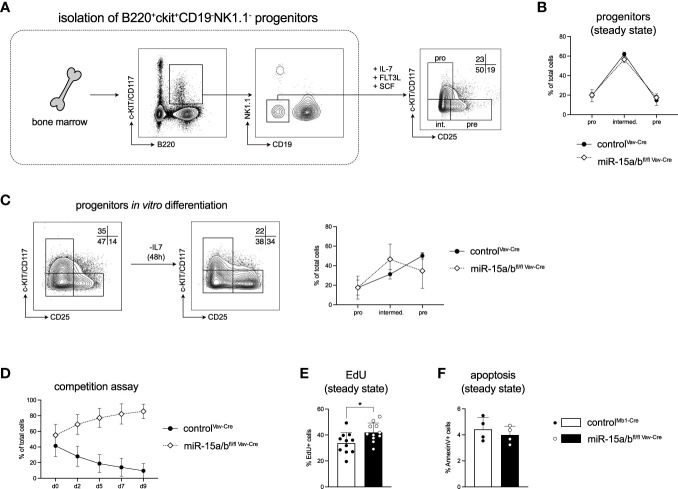
Loss of the miR-15 family results in B cell progenitor expansion *in vitro*. **(A)** Schematic illustration of the sorting strategy for the B cell progenitor *in vitro* culture. B220^+^c-KIT^+^NK1.1^-^CD19^-^ B cell progenitors were sorted from the bone marrow of miR-15a/b^fl/fl Vav-Cre^ and miR-15a/b^+/+ Vav-Cre^ mice and cultured in the presence of IL-7, FLT3L and SCF. A representative FACS plot depicts the percentages of pro-B (c-KIT^+^CD25^-^), intermediate B (c-KIT^-^CD25^-^) and pre-B cells (c-KIT^-^CD25^+^) under basal conditions after 1 week. **(B)** Statistical analysis of the distribution of pro-, intermediate and pre-B cell progenitors derived from miR-15a/b^fl/fl Vav-Cre^ mice and miR-15a/b^+/+ Vav-Cre^ mice under basal conditions. **(C)** Representative FACS blots and statistical analysis of the progenitor B cell differentiation upon IL-7 withdrawal *in vitro.* **(D)** Progenitors from miR-15a/b^fl/fl Vav-Cre^ mice and miR-15a/b^+/+ Vav-Cre^ mice were labeled via stable expression of either dsRed or GFP, mixed at equal ratios at a cell density of 5 x 10^4^ cells/ml and tracked over time by flow cytometry in the presence of all growth factors. **(E)** The bar graph depicts the percentages of EdU^+^ progenitors derived from miR-15a/b^fl/fl Vav-Cre^ mice or miR-15a/b^+/+ Vav-Cre^ mice after labeling with 10 ug/ml EdU for 4 hrs. **(F)** Quantification of the percentage of apoptotic progenitors under steady state conditions as analyzed by AnnexinV staining. Error bars depict the standard deviation of the mean. Numbers indicate the percentage of cells in the respective region. MiR-15a/b^fl/fl Vav-Cre^ progenitors were statistically compared to miR-15a/b^+/+ Vav-Cre^ progenitors by an unpaired two-tailed Student’s t-test. *P < 0.05.

To investigate the role of the miR-15 family in early B cell development and to dissect the molecular mechanism underlying the miR-15-mediated regulation in more detail, we made use of a stromal cell-free *in vitro* system that allows the cultivation of primary B cell progenitors (35). Here, B220^+^c-KIT^+^CD19^-^NK1.1^-^ cells with myeloid and lymphoid potential are sorted from the bone marrow, and in the presence of IL-7, FLT3L and SCF those cells differentiate into CD19^+^ cells that recapitulate all stages of early B cell development including pro-B (c-KIT^+^CD25^-^), intermediate (c-KIT^-^CD25^-^) and pre-B cells (c-KIT^-^CD25^+^; **Figure 3A**). Notably, under excessive growth factor conditions, cells derived from control mice as well as from miR-15a/b^fl/fl Vav-Cre^ mice gave rise to comparable percentages of B cell progenitors *in vitro*, supporting our earlier finding that B cell lineage commitment *per se* is not strongly affected by loss of the miR-15 family (**Figure 3B**). However, a significant advantage of this progenitor culture system is that it also allows an assessment of the differentiation potential of the cells. Upon IL-7 withdrawal as the key factor to instruct proliferation, the steady state distribution of progenitor cells normally shifts from a pro-B/intermediate B towards a pre-B cell-centric pattern (**Figure 3C**). Interestingly, loss of the miR-15 family interfered with this differentiation, as cells derived from miR-15a/b^fl/fl Vav-Cre^ mice failed to generate pre-B cells as efficient as the control cells (**Figure 3C**). As differentiation and proliferation appear to be mutually exclusive phases throughout early B cell development, we wondered whether the reduced differentiation capacity and the elevated progenitor B cell frequencies *in vivo* could be the consequence of enhanced expansion. To test this, actively proliferating miR-15a/b^fl/fl Vav-Cre^ and control progenitor cells derived as described above were transduced with different fluorescent markers, mixed and monitored for the outgrowth of one or the other population. In this assay, miR-15a/b^fl/fl Vav-Cre^ cells consistently outcompeted the control cells over time (**Figure 3D**), pointing towards increased survival, proliferation or both. To further assess this, we quantified EdU incorporation and analyzed apoptosis by AnnexinV staining. While the percentage of apoptotic cells was similar for control and miR-15a/b^fl/fl Vav-Cre^ cells, DNA synthesis as an indirect indicator for proliferation was significantly enhanced upon loss of the miR-15 family (**Figures 3E, F**). Thus, our findings suggest that the miR-15a/16-1 and miR-15b/16-2 clusters limit progenitor B cell proliferation and thereby enable efficient differentiation under physiological conditions.

### The IL-7 receptor is a direct target of the miR-15 family

**Figure 4:**
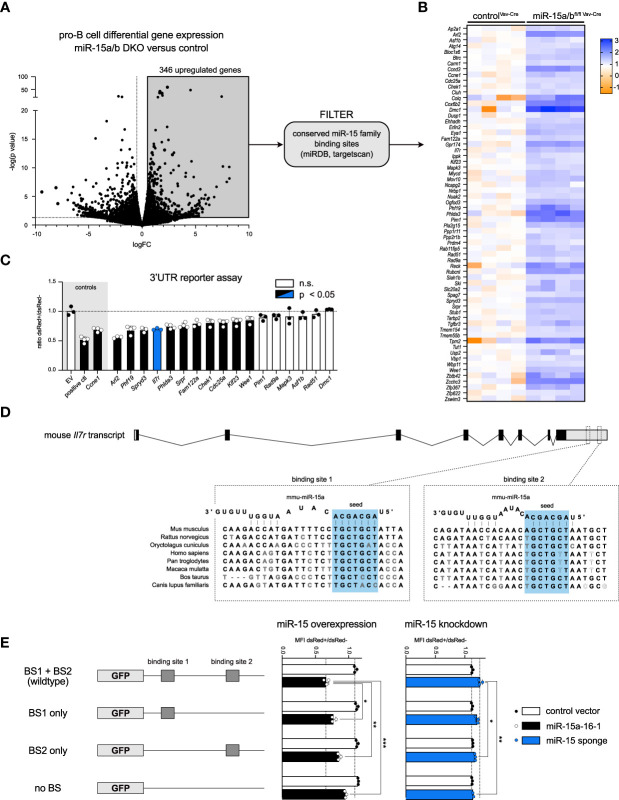
The miR-15 family directly regulates IL-7 receptor expression and cell cycle related genes. **(A, B)** Volcano plot of RNA sequencing data comparing the transcriptome of pro-B cells sorted from miR-15a/b^fl/fl Vav-Cre^ and miR-15a/b^+/+ Vav-Cre^ mice. Significantly upregulated genes (grey quadrant) were filtered for predicted miR-15a, miR-15b and miR-16 binding sites in their 3′‐UTRs using miRDB and targetscan, giving rise to 65 putative miR-15 target genes displayed as a heatmap. Each column corresponds to cells derived from one mouse. **(C)** 3’ UTR assays of a subset of genes identified in **(B)**. A vector lacking a 3′ UTR served as negative control, whereas constructs with a 3′ UTR containing two perfect miR-16 target sites or the 3’ UTR of the already described miR-15 target gene *Ccne1* were defined as positive controls. Error bars depict the standard deviation of the mean. The different groups were compared to the negative control by a one‐way ANOVA followed by Bonferroni testing. **(D)** Schematic illustration of the location and the sequence of the two miR-15 binding sites within the *Il7r* transcript in different species. **(E)** 3’ UTR assay with the *Il7r* sequence or mutants thereof as depicted upon overexpression or knockdown of the miR-15 family. The length of the bars indicates the ratio of GFP MFI comparing transduced and non-transduced cells as measured by flow cytometry. The constructs encoding mutated binding sites were statistically compared to the wild type 3’ UTR construct by an unpaired two-tailed Student’s t-test. Each dot represents the data derived from one independent experiment. *P < 0.05; **P < 0.005; ***P < 0.0005. n.s., not significant.

Having identified a clear function of the miR-15 family in restricting progenitor B cell expansion, we wondered whether this phenomenon could be linked to the repression of particular target genes. With pro-B cells being affected the most in early B cell development, we performed RNA sequencing of sorted control and miR-15a/b^fl/fl Vav-Cre^ cells from this particular stage. Anticipating that direct target genes become upregulated upon deletion of the miR-15 family, we focused on this fraction of the differentially expressed genes (346 genes in total; **Figure 4A**, grey quadrant). Using the well-established targetscan and miRDB algorithms (36–38), this list was further refined by filtering for genes harboring conserved miR-15 binding sites in their 3’ UTRs. This led to the identification of 65 putative target genes including e.g. *Ccne1* (**Figure 4B**, **Supplementary Table S2**), which we have previously described as repressed by miR-15 in B cells, thus validating our strategy (22). Notably and in correspondence with other reports, a GO term analysis with these gene IDs revealed a strong enrichment for cell cycle processes (GO accession 0007049; data not shown). Given that progenitor B cells displayed enhanced proliferation upon miR-15 family loss, we therefore concentrated on genes known to be directly or indirectly involved in cell cycle regulation. To investigate whether this subset of interest encodes mRNAs that could in principle be direct targets of the miR-15 family, we expressed their individual 3’ UTR regions together with GFP as a reporter and quantified reporter fluorescence upon co-expression of the miR-15a/16-1 cluster (**Figure 4C**). This assay revealed miR-15-dependent repression for 11 out of 17 tested candidates, including genes that had been linked to the miR-15 family before, albeit not in context of B cells, as well as novel targets. In addition to *Ccne1*, reporter repression was also prominent for *Cdc25a*, both established drivers of the G1-to-S phase cell cycle transition, suggesting that the miR-15 family limits early B cell development at least in part by directly interfering with cell proliferation.

Beside those targets, our UTR assay also confirmed the direct repression of the *Il7r* gene encoding the alpha chain of the IL-7 receptor (IL7R; **Figure 4C**, blue bar). Notably, IL-7 receptor signaling is well established as a key regulator of early B cell survival, proliferation and differentiation, with both pro- and pre-B cells expressing high levels of the receptor on their surface (39–42). The murine *Il7r* 3’ UTR contains two miR-15 binding sites that are largely conserved across species (**Figure 4D**). To access the functionality of both sites in detail, we mutated the first, the second or both binding sites and performed 3’ UTR reporter assays as described above. In the miR-15 overexpression setting (**Figure 4E**, left panel), loss of either of the two binding sites already increased GFP reporter fluorescence, with a slightly stronger effect for binding site 1, and this derepression was further enhanced once both binding sites were disrupted. For the reciprocal loss-of-function approach, we made use of a miR-15 family-specific sponge construct expressing an mRNA with multiple repeats of a miR-16 binding site, and demonstrated that this sponge is indeed able to sequester endogenous miRNA/RISC complexes in a sequence-specific manner (**Figure S4**). In the context of the *Il7r* 3’ UTR variants, sponge expression resulted in increased reporter fluorescence in the presence of both binding sites (**Figure 4E**, right panel), reflecting that endogenous miR-15 family members bind to and repress the reporter mRNA under steady state conditions. However, this sponge-mediated derepression was reduced when one of the binding sites was disrupted, and was completely abrogated to control levels once both sites were mutated. Together, these experiments clearly indicate that both binding sites individually and additively contribute to miR-15-mediated *Il7r* mRNA repression, and importantly, that this repression manifests under physiological miR-15 expression levels. This raised the question about the functional consequence when this regulatory relationship is disrupted in B cell progenitors.

### Loss of the miR-15 family in B cell progenitors enhances IL7R expression and signaling

**Figure 5:**
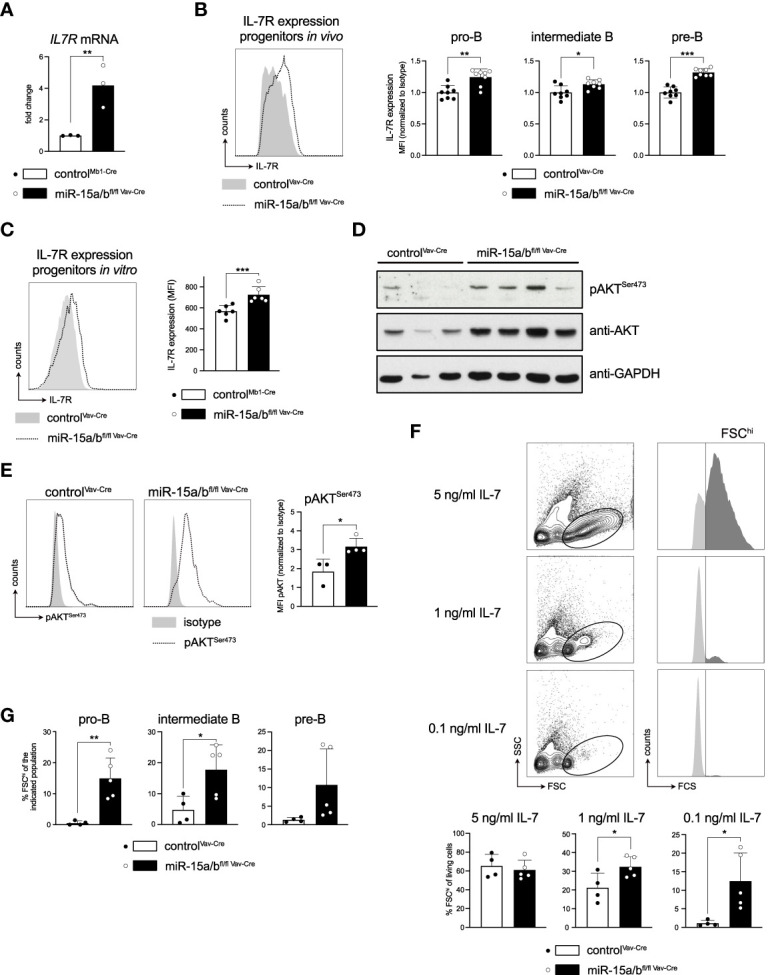
Derepression of the *Il7r* gene increases IL7R expression and promotes downstream signaling. **(A)** IL-7 receptor expression of FACS-sorted pro-B cells (c-KIT^+^CD25^-^) of miR-15a/b^fl/fl Vav-Cre^ and control mice as analyzed by quantitative PCR. **(B, C)** Flow cytometric analysis of IL-7 receptor surface expression in pro- (c-KIT^+^CD25^-^), intermediate- (c-KIT^-^CD25^-^) and pre-B cells (c-KIT^-^CD25^+^) from control or miR-15a/b^fl/fl Vav-Cre^ mice **(B)** as well as *in vitro* cultured progenitors thereof **(C)**. For the *in vivo* data, the MFI values were normalized to the mean IL-7 receptor expression of control mice for each respective experimental day. **(D)** Western blot analysis of control and miR-15a/b^fl/fl Vav-Cre^ progenitors depicting pAKT^Ser473^, AKT and GAPDH protein levels. **(E)** Representative histograms of flow cytometric analysis of intracellular pAKT^Ser473^ levels in control and miR-15a/b^fl/fl Vav-Cre^ progenitors. The bar graph summarizes the pAKT^Ser473^ MFI normalized to the respective Isotype. **(F)** Representative FACS plots depicting the “proliferating” progenitors defined as the FSC^hi^ population (dark grey) upon culturing the cells with 5 ng/ml, 1 ng/ml or 0.1 ng/ml IL-7 for 48 hours. Bar graphs summarize the quantification of the FSC^hi^ population among all living progenitors derived from miR-15a/b^fl/fl Vav-Cre^ or control mice. **(G)** Summary of the percentage of FSC^hi^ cells among pro-, intermediate and pre-B cells upon culturing the progenitors with 0.1 ng/ml IL-7 for 48 hours. MiR-15a/b^fl/fl Vav-Cre^ progenitors were statistically compared to miR-15a/b^+/+ Vav-Cre^ progenitors by an unpaired two-tailed Student’s t-test. *P < 0.05; **P < 0.005; ***P < 0.0005.

Recapitulating our findings from the RNA sequencing, we first used quantitative RT-PCR to confirm that *Il7r* transcripts are indeed upregulated upon loss of miR-15 expression, as suggested by our UTR assays (**Figure 5A**). To investigate whether the increased mRNA levels also translate onto the protein level, thus being functionally relevant, *in vivo* IL-7 receptor surface expression of different B cell developmental stages in the miR-15a/b^fl/fl Vav-Cre^ mice was then analyzed by flow cytometry. Here, all early developmental stages known to be regulated by IL7R signaling, i.e. pro-B, intermediate B and pre-B cells as defined by c-KIT and CD25 surface expression, displayed significantly enhanced IL-7 receptor levels (**Figure 5B**). Anticipating that this increase in receptor expression affects signaling downstream of the IL7R, we again exploited the *in vitro* culture system for primary B cell progenitors as a tool to enable easy cell manipulation and to reduce sample-to-sample variability. Of note, primary progenitors displayed the same increase in IL7R expression as the *in vivo* preparations, justifying our approach (**Figure 5C**).

A key pathway downstream of the IL7R in early B cell development is the PI3K/AKT cascade, which has been demonstrated to mediate proliferation and survival of progenitor cells (42, 43). Investigating activation of this pathway in detail, a Western Blot analysis as well as intracellular flow cytometry demonstrated that both total and phospho-AKT^Ser473^ levels were strongly upregulated in progenitors derived from miR-15 DKO mice compared to controls (**Figures 5D, E**). This raised the question whether increased activation of the PI3K/AKT axis would translate into any functional differences, such as enabling cells to proliferate better or longer under limiting IL-7 concentrations. To address this, control cells and primary miR-15a/b^fl/fl Vav-Cre^ DKO progenitors were cultured with 5 ng/ml, 1 ng/ml and 0.1 ng/ml IL-7 for 48 hrs and cell size as a proxy for proliferation was quantified by flow cytometry (**Figure 5F**). Indeed, while the fraction of large, FSC^hi^ cells was comparable when the growth factor was supplied in excess, limiting IL-7 concentrations significantly favored miR-15a/b^fl/fl Vav-Cre^ DKO cells over the control, basically affecting all populations within the progenitor pool (**Figure 5G**). We conclude that this pro-proliferative phenotype under limiting IL-7 concentrations likely contributes to the observed B cell progenitor expansion *in vivo*.

## Discussion

In this study, we aimed to characterize the early B cell development of mice lacking expression of the miR-15 family, i.e. miR-15a/16-1 and miR-15b/16-2, anticipating that the absence of obvious phenotypes in individual knockout mice is mainly the result of a compensatory effect. This was motivated by our previous *in vitro* study, in which a functional knockdown of the miR-15 family in a pre-B cell line facilitated proliferation and counteracted pre-B to immature B cell differentiation. Mechanistically, this *in vitro* analysis revealed that the miR-15 family exerts its function *via* direct and indirect targeting of the cell cycle regulators cyclin E1 and cyclin D3, respectively (Lindner et al., 2017). In consequence, cells lacking miR-15 activity failed to exit the cell cycle under growth factor limiting conditions, which appeared critical to induce the transcriptional program required for proper differentiation.

Our experiments presented here, exploiting Cre deleter lines specific for the hematopoietic system (Vav-Cre) and for B cells only (Mb1-Cre), largely recapitulate this initial study and demonstrate that the miR-15 family also controls progenitor B cell proliferation and differentiation in a more complex *in vivo* environment. In particular, we show that B cell development in the absence of miR-15a, miR-15b and miR-16 is mainly affected at the pro-B cell stage, in which relative cell percentages were increased in a dose-dependent manner, reaching levels of up to three-fold in the DKO. Our detailed analyses demonstrate that this is mainly due to enhanced proliferation, rather than resistance against apoptosis, resulting in a competitive advantage when compared to wild type progenitor B cells. Pre-B cell percentages and the pre-B to immature B cell ratio were elevated in deficient mice as well, albeit much more pronounced in the B cell specific deletion of the miR-15 clusters, suggesting that both stages are controlled by the miR-15 family under physiological conditions. We argue that the differences in the two Cre strains likely reflect the distinctive kinetics of gene deletion in both systems: Loss of the miR-15 family at an early developmental time-point, such as in the hematopoietic stem cell compartment by Vav-Cre, is fully established once cells enter the pro-B cell stage, and consequently all miR-15 target genes will already be derepressed. We hypothesize that this promotes proliferation and interferes with differentiation of cells at both the pro- and pre-B cell stages, but that the latter phenotype may be partially masked by the pronounced pro-B cell increase. In particular, we propose that a stronger, albeit incomplete block at the pro-B cell stage simply reduces the number of cells that proceed to the next developmental step. Here, fewer pre-B cells may have an advantage to compete for microenviromental factors that promote differentiation, such as CXCL-12, which may in turn weaken the block at the pre-B to immature B cell transition. Indeed, such a model is supported by the phenotype of the single knockouts, which show a milder pro-B cell increase, but already the same pre-B to immature B cell ratio as the double knockout. Mb1-Cre, in contrast, initiates deletion only in pro-B cells and thus at the developmental stage that is mostly affected by loss of the miR-15 clusters. It is therefore tempting to speculate that miR-15-mediated repression in the Mb1-Cre B cell progenitors decreases only gradually, possibly due to the residual presence of mature miR-15 family members even upon disruption of the alleles. Supporting such a model of a delayed knockout, the pro-B cell phenotype in the Mb1-Cre system is much smaller compared to the Vav-Cre-mediated deletion, however, the block at the pre-B to immature B cell transition becomes more pronounced, which recapitulates the phenotype we have previously described with pre-B cell lines *in vitro* (22).

A scenario in which both proliferative stages of early B cell development are controlled by the miR-15 family is not surprising, as pro- and pre-B cells rely on similar signals, mainly derived from the IL-7 receptor. Moreover, the differentiation of both pro- to pre-B and of pre-B to immature B cells is critically dependent on stalling of the cell cycle to enable RAG1/2-mediated VDJ recombination, making a similar regulatory mechanism likely. Supporting this, we found *Ccne1* (encoding cyclin E1) and *Ccnd3* (encoding cyclin D3) to be strongly upregulated upon miR-15 deletion also in pro-B cells *in vivo*, together with other cell-cycle regulators such as *Cdc25a*, all of which have also been described to be derepressed in pre-B or mature B cells lacking miR-15 function (20, 22).

An additional key target gene of the miR-15 family in this context, however, appears to be the *Il7r* gene itself, which we found strongly upregulated on the transcriptional level in pro-B cells and whose enhanced surface expression was then confirmed on all stages of progenitor B cells in the bone marrow. Notably, our *in vitro* study already reported an increased IL7R expression in pre-B cells lacking the miR-15 family, but *Il7r* mRNA levels were not altered in this system (22). In consequence and due to our own selection criteria, we did not consider this gene as a direct target at that time, but have argued for an indirect effect. The discrepancy between these *in vitro* data and our *in vivo* findings may be due to different ratios of posttranscriptional verses translational inhibition of the miR-15 family on the *Il7r* gene, with an almost exclusive translational effect in the pre-B cell lines. That said, the experiments presented here clearly demonstrate a direct repression of the *Il7r* gene, which is in accordance with a T cell-related study (44), and identify two largely conserved binding sites that additively contribute to the posttranscriptional regulation of this gene.

On the functional level, the upregulation of the IL7R upon loss of the miR-15-mediated repression resulted in a clear activation of the PI3K/AKT pathway, indicating that receptor expression is rate-limiting for signal transduction at least under the tested experimental conditions. We therefore conclude, in particular in the light of the well-established pro-proliferative roles of PI3K signaling in B cell progenitors, that the increased IL7R expression drives the accumulation of pro- and pre-B cells in our system, and likely also interferes with proper differentiation as reported before (22). Notably, we have also observed an upregulation of c-KIT, a second growth factor receptor with a critical role in pro-B cells, upon deletion of the miR-15 clusters, and it is conceivable that this enhanced expression also contributes to the pro-proliferative phenotype. Indeed, several *in vitro* studies indicate a stimulatory role of signaling *via* c-KIT in pro-B cells (45–47), however, B cells lacking *c-Kit* gene expression have been shown to develop normally (48). While this points to a non-essential role of *c-Kit* in lymphopoiesis, it does not exclude a positive impact once upregulated, in particular in the context of *c-Kit* being considered as a proto-oncogene in several cancer entities (49). Thus, it remains to be determined whether the increased c-KIT protein levels are a bystander effect without any functional relevance, or whether it in fact aggravates the progenitor B cell phenotype induced by loss of miR-15 family members.

In addition to the derepression of cell cycle regulators, the IL7R and possibly c-KIT, other direct targets of the miR-15 family may be implicated in the pro-proliferative progenitor phenotype as well. *Arl2* (ADP-ribosylation factor 2), for example, the gene with the strongest effect in the 3’ UTR assay due to the presence of four conserved mir-15 binding sites, is involved in ADP/ATP transport in mitochondria and thus plays a role in the cellular energy metabolism. Confirming this gene as a target, loss-of miR-15b has been shown to upregulate Arl2 in cardiomyocytes, resulting in increased cellular ATP levels (50). Moreover, it has been reported that the miR-15-mediated regulation of proliferation and G0/G1 cell cycle arrest is at least to some extent established *via* Arl2 (51). It is thus tempting to speculate that the increased Arl2 expression upon loss of the miR-15 family also elevates ATP levels in B cell progenitors, resulting in enhanced cycling.

Beyond early B cell development and the progenitor B cell phenotype, we observe a slight reduction in mature B cells in Vav-Cre mice both in relative terms as well as absolute numbers, albeit not to a statistically significant extent with respect to the former. We attribute this to a B cell extrinsic effect that is likely secondary to the expansion of the myeloid compartment. In contrast, the mature B cell compartment is not affected upon Mb1-Cre-mediated deletion, which appears the more suitable system to address this point. This indicates that the loss of the miR-15 family – while interfering with proper progenitor B cell differentiation – can be compensated and/or is tolerated on the systemic level, at least with regard to lymphocyte output. We speculate that the surprising absence of a reduction in mature B cells may be explained by two possible, not mutually elusive mechanisms. First, the phenotype may simply correct itself at the mature B cell stage because of the opposing nature of the progenitor B cell phenotype. From our data, we conclude that pro-B and pre-B cell differentiation are compromised upon loss of miR-15 expression, which is indicated by the altered pro-B to intermediate and pre-B to immature B cell ratios in the bone marrow. At the same time, however, miR-15-deficient cells proliferate faster and have a competitive advantage. Thus, it appears feasible that these two phenomena, enhanced proliferation on the one hand and a reduced rate of differentiation on the other hand, balance each other. Relative to the wild type situation, the differentiation rate may be lower, but this is possibly compensated by an increased number of cells that in theory can undergo differentiation, ultimately resulting in a largely unperturbed output of mature B cells. Alternatively, it is conceivable that the developmental block upon miR-15 deletion is so mild that the mature B cell pool is simply filled up to normal levels over a short period of time.

This, of course, raises the question of the physiological function of the miR-15 family. In our study, we have only assessed B cell development under steady-state conditions. Thus, it is possible that the impact of miR-15a/b/16-mediated regulation of pro- and pre-B cell proliferation and differentiation only becomes apparent when the system is challenged. Here, it will be particularly interesting to investigate whether loss of miR-15 promotes or accelerates development of ALL-like leukemia in suitable mouse models. Moreover, loss of the two dominant hematopoietic miR-15 clusters alone may be sufficient to drive progenitor B cell leukemia, although we have not seen signs for this in the relatively small cohorts used for the immunophenotyping. Last, while we have excluded the third cluster of the miR-15 family for this analysis due to the absence of an immune cell-specific phenotype (18), we cannot exclude that miR-497 and miR-195 may partially compensate for the loss of the miR-15a/16-1 and miR-15b/16-2 clusters. It will therefore be important to generate triple knockout mice for all clusters, which may uncover additional aspects of the physiological role of the miR-15 family in B cells.

In the non-B cell population, our study furthermore confirms and reveals additional immune cell compartments that are particularly sensitive to a small decrease in miR-15 levels, as it occurs in either miR15a/16-1 or miR-15b/16-2 single knockout mice, as well as compartments that tolerate deletion of one cluster but are severely affected by the loss of the whole miR-15 family. With respect to the former, we have recapitulated the previous finding that NK cells are dependent on high miR-15 levels, since deletion of the miR-15a/16-1 cluster alone already resulted in a maturation block (Sullivan et al., 2015). Interestingly, loss of the miR-15b/16-2 cluster did not alter NK cell maturation in our hand, but a combined deletion of both clusters further aggravated the developmental block at the stage III to IV transition. We hypothesize that miR-15b/16-2 is simply expressed to a lower extent in this subpopulation, and that its loss fails to reduce miR-15 levels below the critical threshold that provokes a phenotype. The concomitant deletion of both clusters in such a setting, however, completely abolishes miR-15 family expression and consequently enhances the developmental delay.

The myeloid compartment, on the other hand, appears to be less sensitive to changes in miR-15 levels. Here, loss of either cluster is well tolerated, but a complete deletion of both clusters provokes an increase of myeloid cells, in particular neutrophils, in the bone marrow and spleen already in young mice. These findings are in accordance with recent reports describing the onset of acute myeloid leukemia (AML) at an age of 5 months upon combined whole body deletion of miR-15a/16-1 and miR-15b/16-2 (34). This has been linked to deregulated expression of Cyclin D1, Cyclin D2 and anti-apoptotic Bcl-2 (34), but it is likely that additional miR-15 target genes identified or confirmed in this study are involved as well. One interesting candidate in this context is *Cdc25a*, which has already been reported to be regulated by miR-16 in an AML subtype (FLT3-ITD AML) and serves as a critical factor for AML development (Sueur et al., 2020). Similarly, our analysis in B cell progenitors has identified *Fam122a*, encoding an inhibitor of the well-established tumor suppressor protein phosphatase 2A (PP2A), as a direct miR-15 target. Fam122a overexpression has been shown to enhance cell proliferation and colony formation (52), and a recent study even indicates that Fam122a is required for AML growth and survival and that it promotes AML development *in vivo* (53). For the future, it will therefore be interesting to investigate whether these genes are indeed causally linked to AML in the miR-15 DKO mouse model.

Taken together, our data confirm and build on previous findings regarding the role of the miR-15 family in NK cell maturation and myeloid expansion, and establish a novel function in early B cell development *in vivo*. In correspondence with its described tumor-suppressive function, physiological levels of miR-15 family members restrict or limit expansion of progenitor B cells at several stages, thereby ensuring proper transitions between phases of proliferation and differentiation. In analogy to most miRNAs that have been extensively studied so far, the miR-15 family appears to exert this role by modulating an extensive network of target genes, in this case including direct cell cycle regulators as well as prominent growth factor receptors and their downstream signaling cascades. However, more work is needed to elucidate which target genes – both direct and indirect – orchestrate the precise sequence of proliferation and differentiation that is a prerequisite for proper B cell development.

## Data availability statement

The datasets presented in this study can be found in the https://www.ncbi.nlm.nih.gov/geo/ online repository under accession number GSE201238.

## Ethics statement

The animal study was reviewed and approved by animal welfare committees of the University of Veterinary Medicine Vienna and the Medical University of Innsbruck in accordance with good scientific practice guidelines and national legislation (license numbers: BMBWF-68.205/0023-II/3b/2014 and BMBWF-66.011/0021-V/3b/2019).

## Author contributions

KH and SH designed the research, performed experiments, and analyzed data. LA and TR performed experiments. TS analyzed data. KH and SH wrote the first manuscript draft and prepared the figures. KH, AV, TR, and SH reviewed and edited the manuscript. AV, TR, and SH acquired funding. All authors contributed to manuscript revision, read, and approved the submitted version.

## Funding

This work was supported through the Austrian Science Fund (FWF; P30196-B26) to SH.

## Acknowledgments

We thank K. Rossi, S. Gritsch, and I. Gaggl for animal care and their technical assistance.

## Conflict of interest

The authors declare that the research was conducted in the absence of any commercial or financial relationships that could be construed as a potential conflict of interest.

## Publisher’s note

All claims expressed in this article are solely those of the authors and do not necessarily represent those of their affiliated organizations, or those of the publisher, the editors and the reviewers. Any product that may be evaluated in this article, or claim that may be made by its manufacturer, is not guaranteed or endorsed by the publisher.

## Supplementary material

Supplementary Figure 1 Targeting strategy and total cell counts of selected splenic immune cell subsets. **(A, B)** Targeting strategy for deletion of the miR-15a/-16-1 and miR-15b/16-2 cluster. The knock-in alleles were generated by CRISPR/Cas9-induced targeting of murine ES cells with a DNA template containing the miR-15a/16-1 or miR-15b/16-2 clusters flanked by loxP sites (black triangles) and two homology arms. Cre-mediated deletion of the loxP-flanked DNA region creates the miR-15a/16-1 or miR-15b/16-2 null allele, respectively. **(C, D)** Splenic cell numbers were counted and total numbers of the indicated immune cell subsets were calculated accordingly. The bar graphs summarize the total cell counts for each described immune cell subset for miR-15a^fl/fl Vav-Cre^, miR-15b^fl/fl Vav-Cre^, miR-15a/b^fl/fl Vav-Cre^ and miR-15a/b^+/+ Vav-Cre^ mice. Each dot represents the data derived from one mouse. Error bars depict the standard deviation of the mean. Each of the different miR-15 knockout groups were statistically compared to miR-15a/b^+/+ Vav-Cre^ mice by an unpaired two-tailed Student’s t-test. *P < 0.05; *P < 0.005; ***P < 0.0005.

Supplementary Figure 2 Total cell counts of bone marrow immune cell subsets and c-KIT expression in pro-B cells. **(A)** Total bone marrow cell numbers were counted and calculated for the indicated immune cell subsets. The bar graphs summarize the total cell counts for each described subset for miR-15a^fl/fl Vav-Cre^, miR-15b^fl/fl Vav-Cre^, miR-15a/b^fl/fl Vav-Cre^ and miR-15a/b^+/+ Vav-Cre^ mice. **(B)** Representative FACS plots for c-KIT surface expression in control^Vav-Cre^ and miR-15a/b^fl/fl Mb1-Cre^ pro-B cells. The left bar graph depicts the protein expression of c-KIT on pro-B cells relative to a staining with an isotype, the right graph shows *kit* gene expression as determined by quantitative RT-PCR. **(C)** The first bar graph depicts the relative percentages of B cells in the bone marrow of miR-15a/b^fl/fl Mb1-Cre^ and miR-15a/b^+/+ Mb1-Cre^ mice. All other graphs provide total cell numbers, calculated as in A. Each dot represents the data derived from one mouse. Error bars depict the standard deviation of the mean. Each of the different miR-15 knockout groups were statistically compared to miR-15a/b^+/+ Vav-Cre^ mice by an unpaired two-tailed Student’s t-test. *P < 0.05; *P < 0.005; ***P < 0.0005. MFI, mean fluorescence intensity.

Supplementary Figure 3 Hematopoietic lineage progenitors in the bone marrow. **(A)** Schematic gating strategy for flow cytometric quantification of common lymphoid progenitors (CLPs), granulocyte-monocyte progenitors (GMPs), megakaryocyte-erythroid progenitors (MEPs) and common myeloid progenitors (CMPs). **(B)** Representative FACS plots for the respective lineage progenitors in the bone marrow of miR-15a^fl/fl Vav-Cre^, miR-15b^fl/fl Vav-Cre^, miR-15a/b^fl/fl Vav-Cre^ and miR-15a/b^+/+ Vav-Cre^ mice. Numbers indicate the percentage of cells within the respective region. **(C)** Statistical analysis of data as shown in B. Each dot represents the data derived from one mouse. Error bars depict the standard deviation of the mean.

Supplementary Figure 4 Specific derepression of a GFP-based miRNA reporter by a miRNA sponge. WK3 pre-B cells expressing a GFP-based control reporter lacking a miRNA binding site or a miR-16 reporter encoding one site complementary to miR-16 were transduced with a control construct (upper lane) or with a sponge construct encoding multiple binding site for miR-15 family members. Dot plots show the GFP fluorescence of non-transduced (grey) and transduced (light red) cells. Histogram overlays are provided for better visualization. Note that expression of a miR-15-specific sponge derepresses the miR-16 reporter, but not the control reporter.
